# Management of hyperkalemia in patients with kidney disease: a position paper endorsed by the Italian Society of Nephrology

**DOI:** 10.1007/s40620-019-00617-y

**Published:** 2019-05-22

**Authors:** Stefano Bianchi, Filippo Aucella, Luca De Nicola, Simonetta Genovesi, Ernesto Paoletti, Giuseppe Regolisti

**Affiliations:** 1 Nephrology and Dialysis Unit, Department of Internal Medicine, Azienda ASL Toscana Nord Ovest, Livorno, Italy; 2 Nephrology and Dialysis Unit, IRCCS “Casa Sollievo della Sofferenza” Scientific Institute for Research and Health Care, San Giovanni Rotondo, Italy; 3 Division of Nephrology, University of Campania, Naples, Italy; 4 Department of Medicine and Surgery, University of Milano - Bicocca San Gerardo Hospital, Nephrology Unit, Monza, Italy; 5 Nephrology, Dialysis and Transplantation, University of Genoa and Policlinico, San Martino Genoa, Italy; 6 Acute and Chronic Renal Failure Unit, Department of Medicine and Surgery, University of Parma, Parma, Italy

**Keywords:** Hyperkalemia, Chronic kidney disease, Acute kidney injury, Renin–angiotensin–aldosterone inhibitors

## Abstract

Hyperkalemia (HK) is the most common electrolyte disturbance observed in patients with kidney disease, particularly in those in whom diabetes and heart failure are present or are on treatment with renin–angiotensin–aldosterone system inhibitors (RAASIs). HK is recognised as a major risk of potentially life threatening cardiac arrhythmic complications. When an acute reduction of renal function manifests, both in patients with chronic kidney disease (CKD) and in those with previously normal renal function, HK is the main indication for the execution of urgent medical treatment and the recourse to extracorporeal replacement therapies. In patients with end-stage renal disease, the presence of HK not responsive to medical therapy is an indication at the beginning of chronic renal replacement therapy. HK can also be associated indirectly with the progression of CKD, because the finding of high potassium values leads to withdrawal of treatment with RAASIs, which constitute the first choice nephro-protective treatment. It is therefore essential to identify patients at risk of developing HK, and to implement therapeutic interventions aimed at preventing and treating this dangerous complication of kidney disease. Current strategies aimed at the prevention and treatment of HK are still unsatisfactory, as evidenced by the relatively high prevalence of HK also in patients under stable nephrology care, and even in the ideal setting of randomized clinical trials where optimal treatment and monitoring are mandatory. This position paper will review the main therapeutic interventions to be implemented for the prevention, detection and treatment of HK in patients with CKD on conservative care, in those on dialysis, in patients in whom renal disease is associated with diabetes, heart failure, resistant hypertension and who are on treatment with RAASIs, and finally in those presenting with severe acute HK.

## Introduction

HK  is a common finding in patients with kidney disease, due to the effects of kidney dysfunction on potassium (K) homeostasis, and this condition strongly impacts upon the quality of life and prognosis of these patients [1].

Low glomerular filtration rate (GFR) is the main driver for the increase of serum K (sK) above the normal range [2], the main co-determinants being metabolic acidosis, constipation, the typical comorbidities of CKD, diabetes mellitus (DM), heart failure (HF), and the use of RAASIs, the most prescribed cardio-nephroprotective drugs that per se increase sK [3].

HK, besides being associated with fatigue and muscle weakness, remarkably increases the risk of sudden death due to fatal arrhythmias [4], and acts as major driver to start chronic dialysis therapy in patients with end-stage renal disease (ESRD) [5].

The true incidence and prevalence of HK is not known, but it has been estimated to be 2–3% in the general population and 1–10% among hospitalized patients. Individuals with CKD, HF, DM, and those taking RAASIs, as well as more than half of predialysis patients, have an estimated two to threefold higher risk for HK [6].

As for advanced CKD, the prevalence of severe HK is relatively low. In fact, in patients with a GFR < 30 mL/min/1.73 m^2^, severe HK was detected in 1.8% of a large cohort in the US [7], and in approximately 4–5% in a recent Italian study [8]. However, the prevalence of sK > 6.0 mmol/L was reported as high as 34.5% in a recent epidemiological investigation [9].

The clinical relevance of HK has recently been confirmed by a meta-analysis of 27 cohort studies, 10 in the general population, seven in individuals at high cardiovascular risk, 10 in CKD patients, including a total of 1,217,986 subjects followed for an average of 6.9 years. The study demonstrated that: (a) risk of HK onset increased proportionally to GFR decline, starting from GFR < 75 mL/min/1.73 m^2^, and in the presence of pathologic albuminuria (> 30 mg/g); (b) sK > 5.0 mmol/L was an independent predictor of death and ESRD [10].

Despite the epidemiologic dimensions of HK and its negative effects on CKD outcome, current therapeutic strategies are far from optimal. A recent observational study from Italy has confirmed the limits of current approaches to HK even in the setting of renal clinics, i.e. the reference of care for overt CKD [8]. This historical prospective study examined outcomes and determinants in as many as 2443 CKD patients referred to 46 Italian outpatient nephrology clinics. Patients were stratified into four groups by HK status (sK ≥ 5.0 mmol/L) over two visits, basal (referral) and visit after 12 months of nephrology care: “absent” (no–no), “resolving” (yes–no), “new onset” (no–yes), “persistent” (yes–yes).

**Fig. 1:**
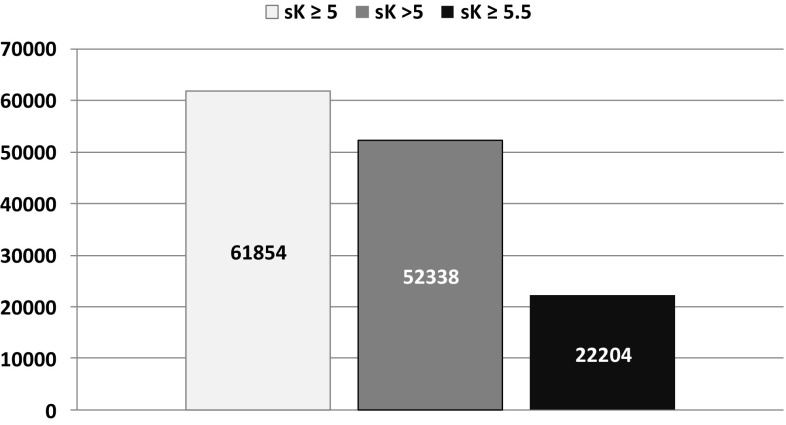
Estimated number of patients with CKD stage 3–5 by hyperkalemia severity followed in Italian nephrology clinics

In this study, mean age was 65 ± 15 years, 58% males, 28% had diabetes, 36% cardiovascular disease, estimated GFR (eGFR) was 35 ± 17 mL/min/1.73 m^2^, proteinuria 0.40 (0.14–1.21) g/day, and RAASIs were prescribed in 79% of the patients. In either visit, mean sK was similar (4.8 ± 0.6 mmol/L) as the prevalence of HK (39% at visit 1 and 37% at visit 2). HK was mild to moderate in the vast majority of cases (sK > 6 mmol/L in less than 4% at either visit). Specifically, after 1 year of treatment in nephrology clinic sK was ≥ 5.0 mmol/L in 39%, > 5.0 mmol/L  in 33% and ≥ 5.5 mmol/L in 14% in overt CKD (eGFR < 60 mL/min/1.73 m^2^). Figure 1 provides a gross estimate of the number of patients with HK followed in Italian nephrology clinics obtained when matching these data with the results of the National Health Survey 2008–2012 on CKD prevalence in Italy, the CARHES (Cardiovascular risk profile in Renal patients of the Italian Health Examination Survey) study [11]. CARHES study disclosed that out of the total number of patients living in Italy with CKD stage 3–5 (*n *=861,835), only 18.4% (*n *=158,600) were aware of their kidney disease and therefore likely to be followed by a nephrologist [11].

When examining the two visits with a 1 year-interval, HK was absent in 46%, resolving in 17%, new onset in 15% and persistent in 22%. Over a median follow up of 3.6 years, start of chronic dialysis and all-cause death occurred in 567 and 349 patients, respectively. The multivariate survival analysis revealed that compared with the reference group (absent), new onset or persistent HK independently increased the risk of dialysis by about 30%. In contrast to an unselected CKD population, mortality did not increase in these patients under stable nephrology care. This finding is expected if one considers the attitude of nephrologists starting dialysis in stage 5 when HK, even when of moderate degree, becomes refractory to medical therapy in order to prevent additional increments of sK potentially associated with fatal arrhythmias [5]. The study therefore suggests that in outpatient nephrology clinics, HK of mild to moderate degree is common (37%) and increases by 30% the need for chronic dialytic treatment.

An additional but important grey area in the management of HK is the treatment of this electrolyte abnormality in dialysis patients. In this setting, sK levels are higher than in non-dialysis CKD, with about 60% and 35% of patients experiencing sK > 5.5 and > 6.0 mmol/L, respectively [9]. HK is more common during the long interdialytic interval and in patients treated with RAASIs to treat underlying cardiovascular disease as in those that need hypertonic dialysate because of poor intradialytic hemodynamic stability [12, 13]. In dialysis patients, the higher the sK values the worse is the prognosis (hospitalization, admission to emergency department and death) [14, 15]. Finally, the optimal management of acute severe HK, which is a true nephrology emergency that requires prompt and efficacious intervention, still remains to be clarified [16].

For the aforementioned reasons, the Italian Society of Nephrology has recently established a working group to develop practical recommendations on the clinical management of HK, in terms of monitoring and treatment. This position statement is the final output of the collaborative work and it is divided into four sections that address the main aspects of HK in CKD population:

1. HK in non-dialytic CKD.
2. HK in dialysis patients.
3. HK in patients with DM, HF and resistant hypertension on treatment with RAASIs.
4. Acute severe HK.

## Hyperkalemia in non-dialytic chronic kidney disease

### Evaluation of the patient with chronic HK

In-depth evaluation of the CKD patients with high sK is the key for graduating intensity of K-lowering treatment. The first objective is to correctly identify patients at high risk of developing more severe and potentially life threatening episodes of HK. Proper assessment is based not only on the exclusion of pseudohyperkalemia (spurious increases of sK usually due to hemolysis of blood samples caused by fist clenching or prolonged use of tourniquet during phlebotomy or delay in processing blood samples), but also on the decision of the correct timing of testing sK. Guidelines suggest measuring sK prior to first prescription or uptitration of RAASIs and in the first two subsequent weeks [17, 18]. Indeed, it is useful monitoring sK over time also independently from RAASIs therapy for three major reasons: (a) CKD per se is a risk factor of developing HK, (b) chance of revealing significant HK increases in parallel with the number of sK tests performed [8], and (c) HK status can change in even one-third of patients over 1-year observation, likely due to the different control of modifiable co-determinants of HK [19] (Table 1).

**Table 1:** Co-determinants of hyperkalemia in CKD

Increased K release from cells
Pseudohyperkalemia
Metabolic acidosis
Absolute or relative insulin deficiency (hyperglycemia), hyperosmolality
Increased tissue catabolism, gastrointestinal bleeding
Non-selective beta blockers and other drugs known to induce hyperkalemia
Reduced urinary K excretion
*K* intake “not adjusted” to GFR level
Reduced aldosterone secretion/effect (diabetes mellitus, RAASIs, K-sparing diuretics)
Reduced distal sodium delivery (heart failure, all-cause oliguria)
Reduced bowel K excretion
Constipation, ileus

*CKD* chronic kidney disease; *GFR* glomerular filtration rate; *K* potassium, *RAASIs* renin-angiotensin-aldosterone system inhibitors

### Position statement 1.1

1. Serum K levels must be measured at the first visit in the Nephrology Unit, as in all subsequent visits, independent of RAASIs prescription.
2. In the presence of elevated or increasing levels of sK, exclude pseudohyperkalemia, extend evaluation to all potential co-determinants of HK and anticipate control visit.

### Target levels of sK

“Clinical normality” of sK can be defined by the range of levels that correspond to the nadir of cardiorenal events attributable to hyper- and hypo-kalemia, thus representing the goal of therapy. This definition must therefore take into account the effect of sK on the global prognosis of the CKD patient. Survival studies in CKD have shown that the relationship between sK and mortality is *U*-shaped, being significant for even mild degree of hypokalemia (sK < 4.0 mmol/L) and hyperkalemia (sK ≥ 5.0 mmol/L) and showing the best survival at sK 4.0–4.5 mmol/L [10, 19].

Therefore, even minimal sK abnormalities should call for attention, because they herald more important changes potentially associated with fatal arrhythmias. The observation is also relevant that in referred CKD, HK significantly increased ESRD risk despite being mild-to-moderate in 94% of cases [8].

### Position statement 1.2

Serum K ≥ 5.0 mmol/L must be considered pathologic in CKD and require careful follow-up and implementation of preventive and therapeutic strategies aimed at maintaining sK in the optimal clinical range (4.0–4.5 mmol/L).

### Nutritional approach to chronic HK

WHO recommends a daily potassium intake of at least 90 mmol or 3.5 g because a lower intake is associated with an increased risk of hypertension and cardiovascular events [20, 21]. These recommendations aimed for the general population may not hold true in CKD patients, due to the intrinsically higher risk of HK. Some studies, including patients with normal or only moderately reduced  GFR, have shown a cardio-nephroprotective effect of higher levels of 24-h urine K excretion, that is proxy of daily K intake [22, 23]. Furthermore, fruit and vegetables, that contain higher amounts of K, also represent the major source of fiber that help in limiting the risk of constipation [22]. This aspect is particularly important in CKD, where fecal loss of K increases with a decline in GFR, as a major compensatory mechanism [24, 25].

While waiting for more and solid evidence on K intake and outcome in mild-to-moderate CKD, it appears useful to maintain a nutritional approach, that is prudent but not restrictive in K intake a priori. On the contrary, patients with advanced CKD should be considered as those under chronic dialysis therapy, that is, they should restrict their daily dietary intake of K (see following paragraph and position statement 2.2).

### Position statement 1.3

At variance with patients with advanced CKD, in non-dialysis CKD of mild-to-moderate degree, restriction of K intake is not recommended unless sK levels are above 5.0 mmol/L in the absence of any other apparent cause; under these latter conditions, it is recommended to limit food with high K content, especially if poor in fibers, and pre-treat (soaking and boiling) before cooking to remove K.

### Pharmacological approach to mild-to-moderate chronic HK

Treatment of mild-to-moderate chronic HK (sK 5.0–6.0 mmol/L) is essentially based on three interventions. The choice and timing of therapy must be tailored to the single patient by taking into account the clinical and laboratory scenario, and eventually integrated with diet modification and downtitration/withdrawal, at least temporarily, of RAASIs: (a) Oral supplementation of sodium bicarbonate (3–5 g/day) is indicated to achieve normokalemia in the presence of concurrent metabolic acidosis (HCO_3_ < 22 mmol/L) [17]. However, bicarbonate appears less effective in reducing sK in patients with advanced CKD [25]. (b) Loop diuretic therapy, either as add-on or uptitration, allows to enhance urinary K excretion. However, this measure is indicated exclusively in the presence of extracellular volume expansion. Indeed, intensive diuretic therapy in CKD requires careful monitoring of patient (body weight, blood pressure and GFR) to prevent hypovolemia and the consequent GFR decline that in turn increases sK [26, 27]. (c) Increase in fecal loss of K by the administration of intestinal K binders. Two binders are currently available, sodium polystyrene sulfonate (SPS) and calcium polystyrene sulfonate (CPS) that are however used in the acute setting and with no evidence on safety and efficacy over the long term, due to the absence of prospective studies or randomized controlled trials (RCTs) on their use as chronic therapy [3]. Furthermore, these two binders must be withdrawn when sK levels drop below 5.0 mmol/L. This therapy therefore is not suitable to enable chronic cardio-nephroprotective therapy with RAASIs, and indeed the two mentioned binders are de facto poorly used in the real world [3].

Two new potassium-binding drugs, namely patiromer and sodium-zyrconium cyclosilicate, not yet available in our country, have undergone extensive clinical testing recently in the setting of chronic hyperkalemia where they have been shown to be effective and relatively safe during long-term administration.

### Position statement 1.4

The pharmacological approach to HK in to mild-moderate chronic CKD is multifactorial and must be based on the specific features of the single patient: (1) Sodium bicarbonate in the presence of metabolic acidosis, (2) Loop diuretics in the absence of euvolemia-hypovolemia and/or low blood pressure, (3) short therapeutic cycles with the two K binders today available (SPS and CPS).

## Hyperkalemia in dialysis patients

### Prevalence and target values of sK in dialysis

Maintenance dialysis remains the main therapy to control K balance in patients with ESRD, especially in the absence of residual renal function. However, HK is quite frequent in the dialysis setting, more in hemodialysis (HD) than in peritoneal dialysis (PD) because of the continuous nature of the latter. The prevalence of HK, when defined as sK > 6 mmol/L, in HD ranges between 10 and 20% in the DOPPS (The Dialysis Outcomes and Practice Patterns Study) survey and DaVita data [28, 29], while in PD it is about 7%, when defined as sK > 5.5 mmol/L [29]. However, a recent study has shown that the overall percentage of HD patients experiencing HK anytime over a 2-year follow-up period can be as high as 74% for sK > 5.1 mmol/L, 58% for sK > 5.5 mmol/L and 35% for sK > 6 mmol/L [9]. On the other hand, more than 25% PD patients have time-averaged sK < 4.0 mEq/L, a level that is associated with a significant increase in mortality risk in this population. Indeed, previous studies have suggested that 10–29% of PD patients require regular K supplements [29]. Differences in reported rates of HK observed between clinical trials are mainly related to heterogeneity in HK definition and patient population, as well as the number of sK tests performed.

K clearance in PD is about 15 ml/min. In patients undergoing 10 L of drainage per day, approximately 35–46 mmol of K is removed. Peritoneal fluids are potassium-free, and K balance is maintained by the continuous nature of the treatment, preserved residual kidney excretion, increased colonic secretion of K, decreased intake of K-rich foods, and transcellular shift driven by insulin release in response to the obligatory glucose absorption from the PD fluids.

HK is the most important electrolyte abnormality in dialysis because it can cause cardiac arrhythmias. Nephrologists try to avoid rapid intradialytic changes of sK by using different models of K removal to limit K gradient between serum and dialysate [30]. However, there is no solid evidence that these approaches improve survival [31]. DOPPS data in HD showed that increased mortality was associated with sK > 5.6 mmol/L [32]; these data have been confirmed in a study where it has been demonstrated that sK > 6.0 mmol/L was associated with an increased risk of morbidity and mortality [14]. The lower mortality rates were observed in hemodialysis with pre-dialysis sK values ranging 4.6–5.3 mmol/L [33], and in PD with sK ranging 4.0–4.5 mmol/L [34].

### Position statement 2.1

Serum K should be kept in the range 4.6–5.3 mmol/L before the beginning of the HD session and 4.0–4.5 mmol/L in PD.

### Management of HK in dialysis

Although K is directly removed from plasma, the distribution of K between the plasma and interstitial fluid is nearly instantaneous; therefore, it is immediately removed from the extracellular fluid. The effect of dialysis on sK concentration depends on the rate of K removal from the extracellular fluid and the rate of K that is replenished from intracellular stores. About 80–140 mmol of K can be removed during a 4-h dialysis session [12], the amount removed depending on the plasma-to-dialysate concentration gradient, blood and dialysate flow rates, and total body K stores. Since replenishment from cellular stores continues when K removal stops, there is a substantial post-dialysis rebound of sK, ranging from 0.5 to 1.0 mmol/L; its concentration gradually rises to pre-dialysis levels within 24 h. Several factors influence dialysis K removal, namely dialysis modality, treatment time, frequency of sessions, residual diuresis, dietary intake, gastrointestinal loss, glycaemia and insulin fluctuations, metabolic acidosis, as well as the use of many drugs, mainly RAASIs [13].

Several HD parameters may affect the magnitude of K removal, including dialysate concentrations of K, bicarbonate and glucose, as well as dialyzer blood flow. The lower the dialysate K concentration, the greater the amount removed, and the lower the final post-dialysis sK. However, the use of low K dialysate may expose patients to potential cardiac side effects. Low K concentration in dialysis bath could be associated with a prolongation of ventricular repolarisation time (expressed by the QT interval of ECG), particularly in presence of low calcium concentration in the dialysate (a). A severe prolongation of ventricular repolarisation time can cause early after-depolarizations that may “trigger” non sustained and sustained tachyarrhythmias, which can lead to ventricular fibrillation. Moreover, low dialysate K concentration is associated with higher intra-dialytic cardiac arrest incidence (b) and both total and sudden cardiac mortality are increased in HD patients with prolonged QT interval duration (c) [35]. On the other hand, patients using dialysate with sK of 3 mmol/L need to be adequately monitored in order to avoid HK. Given that the kidneys are the major route for excretion of dietary K, limiting K intake is critical in functionally anephric dialysis patients. HD patients should restrict their daily dietary K to 2–3 g [36]. Because HK is currently and frequently recognized in the dialysis setting, it is quite clear that current strategies do not allow the full control of HK [9].

Inadequate dialysis, due to erroneous prescription, poor patient compliance or vascular access insufficiency, are additional common risk factors for HK. Therefore, in HK patients it is essential to evaluate dialysis efficiency (modality, dose, treatment time, session frequency, blood and dialysate flow, dialysate K concentration etc.). Generally, standard HD strategies do not allow optimizing HK control [37]. Different schedules often need to be considered such as prolonged dialysis time, alternate-day or daily dialysis [38], or profiled hemodialysis K removal [30]. The latter may be useful in order to avoid high and potentially harmful K gradient between serum and dialysate and post-dialysis K rebound, that are both related to arrhythmias and cardiac arrest [15]. Therefore, we need an adequate mass balance while avoiding excessive changes in serum concentrations. Finally, an additional obstacle to consider in the chronic management of HK in patients on dialysis is the infrequent monitoring of serum K levels, which are generally measured monthly.

### Position statement 2.2

1. When HK is repeatedly detected, dialysis prescription must be re-evaluated: dose, blood and dialysate flow, treatment time and frequency in hemodialysis schedule.
2. In PD: dialysis modality and dialysate volume dwells number.

### When to treat hyperkalemia in dialysis

Opinions vary widely on what level of sK should define “severe” HK and what level constitutes a hyperkalemic emergency [37, 38]; in Table 2 the American Heart Association (AHA)  criteria are reported [39].

**Table 2:** American Heart Association criteria for grading of hyperkalemia

Hyperkalemia grading following American Heart Association [39]		
Mild	Moderate	Severe
sK5.1–5.9 mmol/l	sK6.0–6.9 mmol/l	sK ≥ 7 mmol/l

*sK* serum potassium

Hospital admission is often recommended for patients with sK > 6 mmol/L and electrocardiographic (ECG) monitoring and acute interventions for any patient with sK > 6.5 mmol/L. The ability of ECG features to predict hyperkalaemia of moderate severity is considered poor, since only half of patients with sK > 6.5 mmol/L display typical ECG changes, expecially in the dialysis setting [37, 40].

### Position statement 2.3

1. HD patients should restrict their daily dietary K intake to 2–3 g.
2. In dialysis patients HK must be treated independently of ECG changes.

### How to treat hyperkalemia in dialysis

In dialysis patients, dialysis schedule, dietary intake and concomitant drugs need to be revised. If HK control is still inadequate, K binders need to be considered. Nowadays in Italy two cation exchange resins are available, SPS and CPS. SPS, which exchanges sodium for calcium, ammonium, and magnesium in addition to K, is available since 1950. It is most effective in binding K when it reaches the rectum, either by enema or by oral administration with cathartics.

1000 mg SPS exchanges bound Na for 110–135 mg of K, whereas 1000 mg CPS exchanges bound Ca for 53–71 mg of K. Therefore, the amount of K adsorbed with SPS is expected to be twice that of CPS. SPS exhibits an advantage over CPS because a smaller amount is sufficient to treat hyperkalemia (5–15 g/day). However, if a higher-dose ion-exchange resin is required, physicians should select the type and amount of resin according to the sodium and/or calcium load [41]. Serious gastrointestinal complications from SPS, given with and without sorbitol, have been reported, including fatal colonic perforation and mortality being up to 33% [42]. CKD and ESRD, post-operative or transplant status are the main risk factors [42]. Moreover, when using SPS in dialysis the risk of volume overload needs to be taken into account. Beside being less effective than SPS, CPS also has relevant gastrointestinal side effects such as nausea, with limited tolerability [42]. It is worth noting that these two K binders have not been tested for long-term efficacy and safety.

### Position statement 2.4

Chronic HK in dialysis may be treated with short-term courses of both SPS or CPS.

## Hyperkalemia in patients with heart failure, diabetes and resistant hypertension on treatment with RAAS inhibitors

### Hyperkalemia in patients with diabetes

In clinical practice, HK usually develops as an effect of combination of renal dysfunction and superimposed factors such as HF, high-potassium diet, use of medications inhibiting the RAAS and DM [6].

DM is indeed associated with increased risk of chronic HK, due to blunted insulinemic response to hyperglycemia with reduced K switch to intracellular fluid, plasma hyperosmolality, with enhanced K switch to extracellular fluid, and hyporeninemic hypoaldosteronism, with impairment in K tubular secretion, which plays a major role, especially in type 2 DM.

However, adaptation mechanisms may handle a K load and HK does not ensue in diabetic CKD (DKD) unless a reduction in renal function is present. Accordingly, chronic HK is highly prevalent in patients with DKD. In fact, the prevalence of chronic HK in DM ranges between 8 and 13% [44], but is reported as high as 28% in patients with stage 3 CKD [45].

Also in DKD, the prevalence of chronic HK progressively increases along with increasing number of samplings [46]. This highlights the need for multiple assessment of sK, especially in diabetics with reduced renal function, with the aim to identify subjects at high risk for HK. In most observational studies, together with impaired renal function, chronic use of RAASIs were observed to be independent predictors of the risk of HK in DKD. This is a matter of concern since chronic therapy with RAASIs is strongly recommended by currently available international guidelines for the management of albuminuric DKD at high risk of progression to ESRD [17], since albuminuria reduction was shown to be associated with a significant reduction in the risk of subsequent ESRD [47].

Indeed, the incidence of HK reported in historical trials aimed at evaluating the impact of RAASIs on DKD progression toward ESRD ranged between 18 and 24% with even a 1–2% discontinuation rate from study protocols due to severe HK [48, 49].

Moreover, severe HK together with acute kidney injury (AKI) occurrence was the reason why two large trials exploring the effectiveness and safety of combined RAAS blockade in DKD were prematurely discontinued [50, 51], thus leading to the recommendation of current guidelines not to adopt dual RAASIs therapy in DKD [17, 18]. Despite this, in several studies in DKD patients, the addition of a mineralcorticoid receptor antagonist (MRA) to angiotensin receptor blockers (ARBs) and/or angiotensin convertin enzyme inhibitors (ACEIs), i.e. an actual dual blockade of the RAAS, proved to be effective in reducing albuminuria [52].

### Position statement 3.1

Perform more than one sampling annually for serum K assessment in patients with DKD, and even more frequently in patients administered RAASIs therapy.

Interestingly, a systematic review of 50 RCTs on this topic showed no effectiveness of RAASIs on all-cause mortality in patients with DKD, unless the full or maximum tolerable dose was used, and confirmed that treatment with both ACEIs and ARBs resulted in a significant reduction in the risk of ESRD and of progression from micro- to macroalbuminuria with even a significant increase in regression from micro- to normoalbuminuria [53].

However, current guidelines recommend not to offer RAASIs to CKD patients if their pre-treatment sK is greater than 5.0 mmol/L, and to withdraw therapy when sK increases to 6.0 mmol/L [18].

In fact, HK is associated with increased mortality in the diabetic population and highest rates were observed when CKD coexists [44]. Therefore, in clinical practice, ensuing HK is frequently associated with downtitration or even withdrawal of RAASIs therapy [46], even though this strategy seems to be associated with unfavorable clinical outcome. Interestingly, both submaximal doses of RAASIs and drug discontinuation were shown to be associated with greater incidence of adverse cardiovascular  events and overall mortality in patients with DM, compared to subjects administered the full dose of those medications [54].

Thus, the adequate administration of RAASIs therapy seems to be an important strategy in achieving significant clinical benefit in DKD. It appears that downtitration or even withdrawal of RAASIs for safety reasons (although controlling or even preventing chronic HK) misses the opportunity of offering DKD patients the best available renoprotective therapy.

### Position statement 3.2

Full or maximum tolerable dose of RAASIs medication should be offered to patients with DKD, especially in those with reduced eGFR, in order to maximize nephro- and cardioprotective effects of these drugs.

NICE (National Clinical Guideline Centre, UK) guidelines point out the importance of treatment, and eventually discontinuation of other factors or medications that can induce HK in CKD high risk patients taking RAASIs [18]. They recommend the correction of metabolic acidosis by alkali administration, and adherence to low K diet, in an effort to prevent or offset HK in these high risk patients [18].

Recently, new available agents, such as patiromer and sodium-zirconium cyclosilicate have shown to be effective in controlling HK in DKD patients, with good dose–response and safety profiles [55, 56]. Moreover, in HF patients with reduced renal function and DM, patiromer was effective in steadily maintaining normal sK values, avoiding RAASIs downtitrating and thus allowing full dose administration of spironolactone, aimed at achieving full cardioprotection [57]. Last, new non-steroidal MRAs were shown to be effective in lowering albuminuria in DKD, with a lower incidence of HK, and can be considered a promising therapeutical tool for systematic use in DKD at risk of chronic HK [58].

### Position statement 3.3

In order to offset chronic HK in patients on full or maximum-tolerable dose of RAASIs, it is important to adopt an adequate treatment and nutritional approach to reduce HK. It is also advisable to consider the use of new orally acting agents for chronic administration in DKD patients during effective RAASIs therapy.

In conclusion, DKD is associated with an increased risk of HK, particularly in patients administered RAASIs, in whom more frequent sK assessment is recommended. Downtitration or even withdrawal of RAASIs is associated with worse clinical outcome. This suggests that all therapeutic strategies aimed at lowering sK should be adopted in an effort to improve in the management of this chronic electrolyte disorder mostly in high-risk patients, such as those with DKD, who may benefit from optimal treatment with RAASIs, as recommended by available international guidelines.

### Hyperkalemia in patients with heart failure

Among patients with HF, the prevalence of CKD is high (approximately 41%) and associated with a 50% increase in risk of mortality compared to patients with HF and preserved renal function [59]. The most recent data from the United States Renal Data System [60] show that, among CKD subjects, the percentage of patients with HF is 25.6% compared to a prevalence of 6.1% in the general population. In addition, 40% of patients undergoing HD, 28% of patients on PD and 14% of those undergoing renal transplantation suffer from HF. The 2-year survival of the population with CKD and HF is 65% vs. 75% in patients with HF alone, while ESRD patients with HF have a survival of 66% at 2 years vs. 83% in those without HF [60]. These data underscore the importance of optimally treating CKD patients with HF to reduce the high incidence of mortality.

In the presence of HF, European Cardiology Guidelines [61] recommend ACEIs, along with beta blockers, as first-line drugs, with the addition of MRAs in symptomatic subjects, while ARBs are recommended as an alternative in patients intolerant to ACEIs. In the latest cardiology guidelines, the sacubitril/valsartan (ARNI) combination is recommended in symptomatic patients with HF and reduced ejection fraction. Indications similar to those of European Society of Cardiology (ESC) are those given by the AHA [62] stating that the clinical strategy for the treatment of HF is the inhibition of the RAAS with ACEIs or ARBs or ARNI, in conjunction with beta blockers and with the addition of MRAs in selected patients. Even the US guidelines point out how in patients with HF and reduced ejection fraction ARNI significantly reduces cardiovascular death or HF hospitalization. Both European and US guidelines underline that ACEIs/ARBs should be given with caution to patients with CKD, or elevated serum potassium (> 5.0 mmol/L).

The problem of the occurrence of HK is a crucial point for the treatment of HF in patients, both in the absence and in the presence of CKD. A survey by ESC conducted across 211 Cardiology Centers in 21 countries showed that the presence of HK was a contraindication to the use of ACEIs/ARBs in 8.5% of patients, and that 35% did not take MRAs for the same reason [63]. A US study showed that in a large population of CKD patients with HF only 19% took the maximum dose of ACEIs, while 64% took a submaximum dose and 16% had discontinued the drug [54]. 85% of these patients had at least one episode of HK. Patients taking submaximum doses or who discontinued ACEIs had worse outcomes than patients taking maximum doses of the drug. Similar outcomes were observed between those who were undertreated and those who discontinued the drug [54]. Moreover, even if the AHA defines HK as a sK value > 5.1 mmol/L, a recent study examined the relationship between different levels of sK and mortality among patients with chronic HF and showed that sK above 4.8 mmol/L was also associated with increased mortality risk [64]. If these data were confirmed, the number of HF patients with dangerous values of sK would increase by many units, particularly in the subgroup of subjects with HF and CKD.

The problem of HK as a side effect of therapy in HF patients, already known, ignited after the publication of an important trial, the Randomized Aldactone Evaluation Study (RALES) [65]. The RALES showed that in patients with HF with reduced ejection fraction treated with an ACEI and a loop diuretic and randomized to spironolactone or placebo, there was 30% reduction in the risk of death and 35% reduction in the risk of hospitalization for HF among patients in the spironolactone group. This study excluded patients with a serum creatinine concentration > 2.5 mg/dl. An article published a few years later showed that publication of the RALES study was associated with an increase in the rate of spironolactone prescriptions and in hyperkalemia-associated morbidity and mortality [66]. A post hoc analysis of RALES, aimed at investigating the influence of baseline eGFR and worsening renal function on the efficacy of spironolactone in RALES patients with CKD, showed similar reductions in all-cause of death and hospitalization in patients with a baseline GFR > 60 ml/min/1.73 m^2^ compared to those with baseline GFR < 60 ml/min/1.73 m^2^. However, the risk of HK and renal failure was higher in those with worse baseline renal function, particularly in the spironolactone arm [67].

After RALES, other RCTs demonstrated the efficacy of MRAs in reducing mortality and events in patients with HF [68, 69]. Moreover, in a large sample of hospitalized HF patients with a history of DM and/or CKD, a higher serum creatinine was associated with lower odds of MRAs use. MRAs therapy was associated with lower risk of long-term all-cause readmission, but greater risk of readmission for HK and acute renal failure [70].

Recently, data from a sub-analysis of the TOPCAT study, a RCT aimed at evaluating the efficacy of spironolactone vs. placebo in a population of patients with HF and preserved ejection fraction were published [70]. The authors evaluated the association between baseline renal function and the efficacy and safety of the drug over a 4-year follow-up period. Spironolactone was effective in reducing the incidence of death, aborted cardiac arrest and hospitalizations across three categories of eGFR (> 60, 45–60, < 45 ml/min/1.73 m^2^), but the drug was also associated with a higher risk of hyperkalaemia, worsening of renal function and drug discontinuation compared to placebo in the lower eGFR categories [71].

The latest cardiology guidelines included for HF the treatment with an ARNI. The ARNI treatment was compared with enalapril in HF patients in addition to optimal therapy. Patients with an eGFR below 30 ml/min/1.73 m^2^ were excluded and the mean creatinine plasma value was 1.1 mg/dl for both arms. ARNI was superior to enalapril in reducing the risk of death and of hospitalization for HF. The rate of patients with elevated serum potassium (> 5.5 mmol/l) was similar with both therapies, while the rate of patients with serum potassium > 6.0 mmol/L was significantly higher in enalapril group [72].

In conclusion, it is clear that even HF patients with CKD can benefit from the use of ACEIs, ARBs and MRAs. However, it is also evident that this population is more at risk for the occurence of HK, a potentially very dangerous complication in terms of hospitalization and mortality. Providing nephrologists with the necessary tools to enable them to control the onset of HK should significantly improve the prognosis of CKD patients with HF.

### Position statement 3.4

1. The drugs that the cardiology guidelines recommend for the treatment of patients with HF are effective in reducing adverse events, re-hospitalizations and mortality, even in patients with the simultaneous presence of HF and CKD.
2. Therefore, there should be no difference in the therapeutic approach in patients with HF with and without CKD.
3. ACEIs, ARBs and MRAs are among the drugs suggested by cardiology guidelines as first-line treatment.
4. All of these classes of drugs can lead to hyperkalemia, particularly in patients with reduced GFR and those experiencing a worsening of renal function.
5. In HF patients with CKD, special attention should be paid to the monitoring of sK and renal function. In these patients, all dietary and pharmacological measures should be implemented to prevent and control increases in sK, before reducing or discontinuing ongoing therapies.
6. In HF patients with CKD who are not taking RAASIs therapy because of high sK values, all dietary and pharmacological measures should be implemented to reduce sK and allow them to receive appropriate treatment for their cardiological disease.

### Hyperkalemia in patients with resistant hypertension

Resistant hypertension (RH) is defined as hypertension when the recommended treatment (at least three drugs, including a diuretic) strategy fails to lower systolic (SBP) and diastolic blood pressure (DBP) values to < 140 mmHg and/or < 90 mmHg and the inadequate control of BP is confirmed by ambulatory blood pressure and home blood pressure monitoring. Resistant hypertension is associated with a poor prognosis. The PATHWAY-2 randomized trial performed in 314 patients with RH assessed the role of plasma aldosterone, renin, and aldosterone-to-renin ratio (ARR) as predictors of home SBP and the effect of amiloride on lowering clinic SBP [73]. The study demonstrated that SBP reduction by spironolactone was predicted by ARR and plasma renin values. Amiloride significantly reduced SBP, not different from spironolactone [73]. The results suggest that RH is commonly a salt-retaining state, most likely due to inappropriate aldosterone secretion. In accordance with these findings, in recent years, an increasing body of evidence has shown benefit of MRAs, such as eplerenone and spironolactone, in improving BP control in patients with RH. A recent meta-analysis based on data from multiple RCTs evaluated the efficacy of add-on use of spironolactone in patients with RH. The antihypertensive effects were assessed in 869 patients included in four trials. The reduction in SBP and DBP in patients treated with spironolactone was greater than that observed in the placebo group. The rate of serious adverse effects or patient withdrawal from the trials tended to be higher in patients treated with spironolactone than placebo [74].

CKD is often present in RH subjects. In a population of 37,061 presenting with uncontrolled hypertension despite taking ≥ 3 drugs, CKD was associated with a higher risk for RH [75]. Data from 17,466 patients, 1576 of which had RH, showed a CKD prevalence of 20.4% [76]. In a small sample (*n *=436) of hypertensive CKD patients, 22.9% (*n *=100) were classified as true resistant [77] and in a larger population of RH patients (*n *=7436), 24.0% had CKD (eGFR < 60 ml/min/1.73 m^2^) [78].

The use of MRAs for hypertension treatment is limited in patients with moderate-to-severe CKD, mainly due to the risk of HK. At present, only studies performed in a small number of patients evaluated the efficacy and safety of MRAs in CKD patients with RH. In 36 patients with stage 3 CKD MRAs induced a significant decrease in SBP and DBP. However, a concomitant significant increase in sK and serum creatinine was observed (one case of acute renal failure and three cases of significant hyperkalemia) [79]. In 41 patients with GFR between 25 and 50 mL/min and RH, randomly divided into two groups (placebo or spironolactone), there was a significant decrease in SBP and DBP after 6 and 12 weeks in the patients who received spironolactone, while there was no change in BP in the control group. HK occurred in one subject in the spironolactone group [80].

Recently, the rationale and design of a RCT have been published that will evaluate if the potassium-binding patiromer used concomitantly with spironolactone could prevent HK and allow spironolactone use for the management of hypertension in CKD patients (eGFR 25 to  45 mL/min/1.73 m^2^) with RH. The endpoints are the differences in the proportion of patients remaining on spironolactone and in SBP values between the two groups (spironolactone and placebo) after 12 weeks of treatment [81].

The latest guidelines from the European Society of Hypertension [82] established that, besides optimal doses or best-tolerated doses of an optimal therapy, typically including ACEIs or ARBs along with a calcium channel blockers and a thiazide diuretic, the fourth-line treatment should involve a blockade of aldosterone through the use of MRAs (spironolactone up to 50 mg/day). However, it is suggested that the use of spironolactone be restricted to patients with an eGFR > 45 mL/min/1.73 m^2^ and a sK concentration < 4.5 mmol/L. The AHA [83] strongly suggests that the management of RH include lifestyle interventions, use of thiazide-like diuretics and the addition of a MRA. For these reasons, is important to provide the nephrologist with tools to allow them to apply the cardiology guidelines in patients with CKD and RH, at high risk of developing hyperkalemia, if simultaneously treated with RAASIs and a MRA.

### Position statement 3.5

1. In the presence of RH, the use of MRAs on top of antihypertensive therapy commonly including a calcium channel blocker, an ACEI/ARB, and a diuretic is indicated. Data on the use of MRA in CKD patients with RH are few, but suggest that even in patients with CKD MRAs reduce BP and urinary excretion of albumin.
2. Given the frequent occurrence of hyperkalemia, close monitoring of plasma potassium and renal function is important in patients with CKD and RH, particularly if they are simultaneously treated with ACEIs/ARBs and MRA.
3. Attention should be even greater in patients with an eGFR < 45 ml/min/1.73 m^2^. In these patients all dietary and pharmacological measures should be implemented to prevent and control increases in sK, before reducing or discontinuing ongoing therapies.
4. In RH patients with CKD and eGFR < 45 ml/min/1.73 m^2^ not taking ACEIs/ARBs and MRA, all dietary and pharmacological measures should be implemented to reduce plasma K values and allow them to receive appropriate treatment for their hypertensive disease.

## The treatment of severe hyperkalemia

### Relevance of the problem

Severe HK is a potentially lethal condition, and represents a clinical emergency. With few exceptions (e.g., tumor lysis syndrome or rhabdomyolysis), it is associated with oliguria and low GFR, either in the setting of AKI or advanced CKD.

Indeed, HK has been listed as one of the most common complications of AKI for several decades [84, 85]. In a recent retrospective study enrolling > 18,000 adults admitted to intensive care units, the frequency of HK increased from 8.8% in patients with AKI stage 1 to 32.2% in those with AKI stage 3 [86]. In a series of 923 consecutive hospitalized adult patients with at least one episode of severe HK (defined as a sK concentration of > 6.5 mmol/L), AKI was a coexisting condition in 22.2% of the patients with normal baseline function and in 51.8% of those with underlying CKD [87].

HK is associated with increased mortality in both AKI [86] and CKD [8]. Moreover, 3–5% of deaths in patients with ESRD on routine HD may be attributed to HK [88]. Severe bradyarrhythmias and complex ventricular tachyarrhythmias are life-threatening dangers associated with HK, especially if interfering drugs (e.g. digoxin) and/or severe acidosis are also present [89, 90]. ECG alterations are common in hyperkalemic patients [91] and are more frequently present when sK increases rapidly [92], ranging from peaked T-waves to QRS widening, disappearance of P-wave, and to advanced atrioventricular blocks, wide-QRS tachyarrhythmias and asystole in severe HK. Although there is a loose relationship between levels of sK and ECG changes in hyperkalemic patients, the ECG sensitivity does not exceed 34–43% as a unique tool to detect HK [91], and may also be misleading [93, 94]. Aspecific symptoms may be present with or without ECG changes, including gastrointestinal symptoms, paresthesias, decrease of deep tendon reflexes, and weakness progressing to paralysis [90, 92].

### What are the treatment options?

If HK is a casual laboratory finding and seems unjustified on clinical grounds in a given patient, pseudohyperkalemia should be ruled out before starting treatment [37, 92]. In all other cases, even if the patient is not clinically unstable, immediate evaluation of treatment options is mandatory. While the presence of serious ECG changes or arrhythmias are most relevant with respect to treatment approach, the absolute levels and rate of increase in sK [92], as well as urine output and potential hidden sources that maintain high sK, are also important factors that must be taken into account.

If the patient is severely oliguric or anuric (e.g. in the setting of ESRD or AKI stage 3), there is ongoing K entry in the extracellular compartment (e.g. rhabdomyolysis or large hematomas), the patient is volume-overloaded and loop diuretics are unlikely to be effective, emergency dialysis should be performed [37, 92]. If the patient has ESRD and a functioning vascular access, dialysis should be started immediately, irrespective of the presence of ECG alterations [40]. Whilst in this case the time interval between patient assessment and dialysis start is minimized, a variable delay is expected if a central venous catheter must be inserted in a patient with AKI. In this latter case, urgent treatment must be initiated to reduce the risk of life-threatening arrhythmias.

### Position statement 4.1

1. If the patient is severely oliguric or anuric (e.g. in the setting of ESRD or AKI stage 3), there is ongoing K entry in the extracellular compartment (e.g. rhabdomyolysis or large hematomas), the patient is volume-overloaded and loop diuretics are unlikely to be effective, emergency dialysis should be performed.
2. If the patient has ESRD and a functioning vascular access, dialysis should be started immediately, irrespective of the presence of ECG alterations.
3. If a central venous catheter must be inserted in a patient with AKI, urgent medical treatment must be initiated to reduce the risk of life-threatening arrhythmias.

When dialysis is started, profiling of dialysate K concentration to maintain a constant gradient between K  dialysate concentration and sK seems preferable, compared to a constantly low (i.e. < 2 mmol/L) K concentration in the dialysis bath [95]. In fact, retrospective analyses suggest that low K dialysate concentration may be associated with sudden cardiac death [96], although there are no prospective data showing a favorable effect of changing K dialysate concentration on the incidence of sudden cardiac death. A rebound 0.1–1.0 mmol/L increase in sK after the end of the dialysis session should be taken into account [37, 92].

When electrocardiographic signs of HK are detected, the first treatment step is directed at stabilizing cell membrane potential. Immediate slow intravenous administration of calcium salts, either calcium gluconate or calcium chloride, must be performed to contrast the ongoing cell membrane depolarization [37, 92]. In fact, calcium re-establishes a larger transmembrane voltage gradient within a few minutes of intravenous administration [92], possibly via restoration of rapid sodium channel Nav1.5 functionality [97]. Calcium chloride contains 13.6 mEq per 10 mL, whilst calcium gluconate contains 4.6 mEq per 10 mL. Calcium chloride may cause tissue necrosis on extravasation if given through a peripheral vein, and thus calcium gluconate is generally preferred, unless a central venous access is available, or the patient has cardiac arrest [97]. One ampule of 10% calcium gluconate should be infused over 1–2 min, and can be repeated after 5 min if ECG does not improve (Table 3). Calcium salts should be used with caution if digitalis toxicity is suspected [92].

**Table 3:** Emergency pharmacological treatment of severe hyperkalemia

Treatment	Expected decrease in sK	Onset of action	Duration of effect	Side effects/risks
10% calcium gluconate 1 ampule (1 g) by slow (1–2 min) bolus (may be repeated after 5 min)	None	< 3 min	20–50 min	Caution/avoid if digitalis toxicity strongly suspected
Regular insulin 0.1 IU/Kg BW (up to max 10 IU) by i.v. bolus, preceded by (if serum glucose <250 mg/dL) 50% dextrose 50–100 mL (25–50 g) or	0.6–1.2 mEq/L after 1 h	15 min	4 h	Hypoglycemia (up to 30% in patients with advanced CKD)
Regular insulin 20 IU by i.v. infusion over 1 hour together with i.v. infusion of dextrose 60 g	0.60–0.92 mEq/L after 1 h			Hourly monitoring of serum glucose concentration for at least 3 hours necessary
10–20 mg nebulized salbutamol (20 gtt of a 0.5% salbutamol solution repeated up to 8 times in 120 min) or	0.53–0.98 mEq/L	Within 30 min	Maximum effect at 90 min	Tremor, tachycardia, palpitations, anxiety More effective if used in conjunction with insulin/dextrose Use with caution in patients with heart disease 12–40% of patients unresponsive, especially if on treatment with betablockers Avoid in patients with ischemic heart disease
0.5–2.5 mg i.v. salbutamol	0.87–1.4 mEq/L		Maximum effect at 30 min	
1.4% (1/6M) or 8.4% (1 M) sodium bicarbonate by i.v. infusion, 10–20 mEq/h	Variable (up to 2 mEq/L after 10 mEq/L of increase in serum bicarbonate concentration in acidemic patients with CKD			Use only in acidemic patients Risk of hypernatremia, volume overload, tetany, and pCO2 increase in patients with respiratory failure
Furosemide 1 mg/Kg as i.v. bolus (up to 80 mg), followed by 10 mg/h continuous infusion	Unpredictable	15 min	Duration of infusion	Use only in hypervolemic patients May be combined with thiazides or thiazide-like diuretics
Sodium polystyrene sulphonate 30 g with 100 ml 20% sorbitol orally or by rectal enema	Doubtful efficacy in the acute setting	At least 2 h	6 h	Risk of colonic necrosis (low) Bowel obstruction must be ruled out before administration Not recommended as a first-line treatment of emergency hyperkalemia

*BW* body weight, *CKD* chronic kidney disease, *pCO*_*2*_ partial pressure of carbon dioxide, *sK* serum potassium

As the administration of calcium salts will not decrease sK, the following step to be undertaken is to force intracellular translocation of K. Stimulation of the Na/K ATPase activity and GLUT4 receptor recruitment by the intravenous administration of insulin and glucose is the most efficient way to induce transcellular K redistribution [37, 92]. While an intravenous bolus of 10 units of regular insulin together with an intravenosus bolus of 25–50 g of dextrose is indicated as a standard approach if serum glucose concentration is < 250 mg/dL [92], hypoglycemia may ensue, especially in patients with CKD [98]. Thus, a reduced (i.e. 5 units vs. 10 units) dose of regular insulin [99] or a weight-based intravenous insulin dose (0.1 units/Kg up to a maximum 10 units) [100] have been advocated as preferential protocols to treat severe HK in patients with or without CKD/ESRD. The use of short-acting vs. regular insulin has also been investigated, but deserves further studies [92]. A recent systematic review recommended either the continuous infusion of 20 units of regular insulin over 1 h with 60 g of dextrose or an intravenous 10 units regular insulin bolus together with 50 g of dextrose to reduce the risk of hypoglycemia in patients with severe HK [43]. A treatment scheme with insulin/dextrose for severe HK is summarized in the Table 3.

The administration of nebulized or intravenous salbutamol is another strategy to decrease sK in severely hyperkalemic patients (Table 3), especially when this treatment is combined with insulin/dextrose [37, 92]. The salbutamol dose that promotes the intracellular translocation of K via stimulation of Na/K ATPase (i.e. 10–20 mg if given by nebulization, or 0.5–2.5 mg if given intravenously) is 4–8 times higher than that used to treat bronchospasm. Side effects (e.g. tremor, palpitations, anxiety) are usually mild and are more frequent with intravenous administration, but arrhythmias may develop in susceptible patients with heart disease [37]. Moreover, efficacy may be reduced in patients treated with non-selective betablockers [37, 92].

The intravenous administration of sodium bicarbonate may theoretically cause transcellular K redistribution through the stimulation of transmembrane H/K exchange, Na/H exchange, sodium-bicarbonate cotransport and Na/K ATPase [37, 92]. However, the efficacy of sodium bicarbonate administration, particularly in the form of isotonic formulations or combinations with dextrose/insulin, is mainly seen in acidemic patients [37, 92]. The administration of large amounts of isotonic or hypertonic bicarbonate may also induce hypernatremia and fluid overload, and may increase pCO_2_ in patients with respiratory insufficiency [101]. Thus, the administration of sodium bicarbonate should not be advised as a first-line strategy in severe HK, and should be given preferentially as part of combination therapy in patients with metabolic acidosis (Table 3).

The intravenous administration of loop diuretics, possibly combined with thiazides or thiazide-like diuretics and acetazolamide, may be considered in hyperkalemic and hypervolemic patients with only moderately compromised renal function [92].

Finally, the oral or rectal administration of SPS and CPS together with sorbitol can be performed with the aim of increasing K  elimination in the distal colon, provided that bowel obstruction has been ruled out. However, the minimum 2-h time-lag before effect onset, variable efficacy, and the small though significant risk of colonic necrosis [37, 92, 101] (Table 3) do not support the administration of SPS as part of the treatment of patients with acute severe HK. New-K-binding drugs, namely patiromer and sodium–zyrconium cyclosilicate have undergone extensive clinical testing recently in the setting of chronic HK, and have proved to be effective and relatively safe in the short term [102]. In particular, sodium–zyrconium cyclosilicate  was shown to be able to decrease sK by  0.4 mmol/L at 1 h and 0.7 mmol/L at 4 h after the oral administration of a 10 g dose in 45 patients with baseline sK ranging between 6.1 and 7.2 mmol/L. Moreover, sK was < 6.0 mmol/L in 80% of the patients, and < 5.5 mmol/L in 52% by 4 h [103]. While more data are awaited, sodium–zyrconium cyclosilicate may be interesting in the acute treatment of severe hyperkalemia, due to its rapid effect onset and K binding along the entire intestinal tract.

**Fig. 2:**
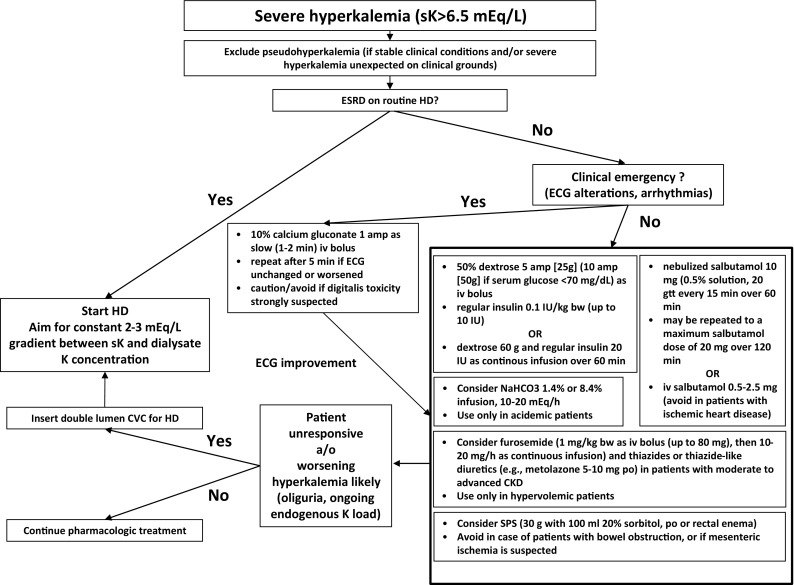
Algorithm for the emergency treatment of severe hyperkalemia

An algorithm for the emergency treatment of severe HK is shown in Fig. 2.

### Position statement 4.2

1. If ECG signs of hyperkalemia are detected, the first treatment step is directed at stabilizing cell membrane potential. Immediate slow intravenous administration of calcium salts, either calcium gluconate or calcium chloride, must be performed to contrast the ongoing cell membrane depolarization.
2. As the administration of calcium salts will not decrease sK, the following step to be undertaken is to force intracellular translocation of potassium. The intravenous administration of insulin and glucose is the most efficient way to induce transcellular potassium redistribution.
3. The administration of nebulized or intravenous salbutamol is another strategy to decrease sK in severely hyperkalemic patients, especially when this treatment is combined with insulin/dextrose.
4. The intravenous administration of sodium bicarbonate may theoretically cause transcellular potassium redistribution. However, the efficacy of sodium bicarbonate administration, particularly in the form of isotonic formulations or combinations with dextrose/insulin, is seen mainly in acidemic patients.
5. The administration of large amounts of isotonic or hypertonic bicarbonate may also induce hypernatremia and fluid overload, and may increase pCO_2_ in patients with respiratory insufficiency [100]. Thus, the administration of sodium bicarbonate should not be advised as first-line strategy in severe hyperkalemia, and should be given preferentially as part of combination therapy in patients with metabolic acidosis.
6. If hemodialysis can be started immediately in a patient with a functioning vascular access (e.g., arteriovenous fistula or central venous catheter), pharmacologic strategies to force transcellular potassium redistribution should not delay dialysis start, and may be foregone as they may also, on theoretical grounds, decrease potassium removal by dialysis.
7. The intravenous administration of loop diuretics, possibly combined with thiazides or thiazide-like diuretics and acetazolamide, may be considered in hyperkalemic and hypervolemic patients with only moderately compromised renal function.
8. The oral or rectal administration of SPS or CPS together with sorbitol can be performed with the aim of increasing fecal potassium elimination in the distal colon, provided that bowel obstruction has been ruled out. However, the minimum 2-h time-lag before effect onset, variable efficacy, and the small though significant risk of colonic necrosis do not support the administration of SPS or CPS as part of the treatment of patients with acute severe hyperkalemia.

## Conclusion

The management of HK is a major yet unmet need in the Nephrology setting, in spite of the negative impact of this alteration on patient prognosis and the obvious high costs of hospitalization and dialysis [104].

Although clinically challenging, the treatment of severe acute HK is sufficiently outlined, as in patients on dialysis. On the contrary, the treatment of chronic HK in patients with CKD still represents a therapeutic challenge, because its detection is often the cause of suboptimal medical treatment, responsible for faster progression of CKD, and an earlier recourse to renal replacement therapy. Thus, there is an important need for a well-tolerated and effective treatment, allowing the control of sK in a safe range, in patients with CKD or cardiovascular disease who require the therapeutic advantage of treatment with RAASIs, as in patients with ESRD still on conservative therapy.

The Italian Society of Nephrology, being well aware of the clinical burden of HK and the limits of chronic therapy, suggest that nephrologists devote a high level of attention to patients with even a moderate increase in sK and suggest the prompt implementation of the two new K binders approved by Food and Drug Administration in US and the European Medicines Agency with efficacy and safety proven by controlled randomized trials in CKD [56].

Indeed, both K binders (patiromer and sodium–zyrconium cyclosilicate) have demonstrated therapeutic efficacy in lowering sK, associated with a satisfactory tolerability and safety profile. Numerous clinical randomized trials have shown that the use of these drugs in patients with CKD, also in those with concomitant HF and/or DM, allows a long-term effective control of sK, allowing the maintenance of efficacious dosages of RAASIs, indicated with high level of evidence from the nephrology and cardiology guidelines as the most effective therapeutic strategy to slow the progression of CKD and to improve the prognosis of patients with heart failure [105].

## References

[1] Moranne O, Froissart M, Rossert J (2009). Timing of onset of CKD-related metabolic complications. J Am Soc Nephrol.

[2] Gumz ML, Rabinowitz L, Wingo CS (2015). An integrated view of potassium homeostasis. N Engl J Med.

[3] Kovesdy CP, Appel LJ, Grams ME (2017). Potassium homeostasis in health and disease: a scientific workshop cosponsored by the national kidney foundation and the american society of hypertension. J Am Soc Hypertens.

[4] Faxén J, Xu H, Evans M (2019). Potassium levels and risk of in-hospital arrhythmias and mortality in patients admitted with suspected acute coronary syndrome. Int J Cardiol.

[5] van de Luijtgaarden MWM, Noordzij M, Tomson C (2012). Factors influencing the decision to start renal replacement therapy: results of a survey among European nephrologists. Am J Kidney Dis.

[6] Sarafidis PA, Blacklock R, Wood E (2012). Prevalence and factors associated with hyperkalemia in predialysis patients followed in a low-clearance clinic. Clin J Am Soc Nephrol.

[7] Luo J, Brunelli SM, Jensen DE, Yang A (2016). Association between serum potassium and outcomes in patients with reduced kidney function. Clin J Am Soc Nephrol.

[8] Provenzano M, Minutolo R, Chiodini P (2018). Competing-risk analysis of death and end stage kidney disease by hyperkalaemia status in non-dialysis chronic kidney disease patients receiving stable nephrology care. J Clin Med.

[9] Rossignol P, Lamiral Z, Frimat L (2017). Hyperkalaemia prevalence, recurrence and management in chronic haemodialysis: a prospective multicentre French regional registry 2-year survey. Nephrol Dial Transplant.

[10] Kovesdy CP, Matsushita K, Sang Y (2018). Serum potassium and adverse outcomes across the range of kidney function: a CKD Prognosis Consortium meta-analysis. Eur Heart J.

[11] De Nicola L, Donfrancesco C, Minutolo R (2015). Prevalence and cardiovascular risk profile of chronic kidney disease in Italy: results of the 2008–2012 national health examination survey. Nephrol Dial Transplant.

[12] De Nicola L, Bellizzi V, Minutolo R (2000). Effect of dialysate sodium concentration on interdialytic increase of potassium. J Am Soc Nephrol.

[13] Movilli E, Camerini C, Gaggia P (2018). Use of renin-angiotensin system blockers increases serum potassium in anuric hemodialysis patients. Am J Nephrol.

[14] Brunelli SM, Du Mond C, Oestreicher N (2017). Serum potassium and short-term clinical outcomes among hemodialysis patients: impact of the long interdialytic interval. Am J Kidney Dis.

[15] Brunelli SM, Spiegel DM, Du Mond C (2018). Serum-to-dialysate potassium gradient and its association with short-term outcomes in hemodialysis patients. Nephrol Dial Transplant.

[16] Abuelo JG (2017). Treatment of severe hyperkalemia: confronting 4 fallacies. Kidney Int Rep.

[17] Kidney international supplements|KDIGO 2012 clinical practice guideline for the evaluation and management of chronic kidney disease|sciencedirect.com. http://www.sciencedirect.com/journal/kidney-international-supplements/vol/3/issue/1. Accessed 21 Jan 2018 10.1038/ki.2013.243 23989362

[18] National Clinical Guideline Centre (UK) (2014). Chronic kidney disease (partial update): early identification and management of chronic kidney disease in adults in primary and secondary care.

[19] De Nicola L, Di Lullo L, Paoletti E (2018). Chronic hyperkalemia in non-dialysis CKD: controversial issues in nephrology practice. J Nephrol.

[20] WHO (2012). Guideline: potassium intake for adults and children.

[21] Hoorn EJ, Vogt L, Rotmans JI (2018). Nutritional management of chronic kidney disease. N Engl J Med.

[22] Cupisti A, Kovesdy CP, D’Alessandro C, Kalantar-Zadeh K (2018). Dietary approach to recurrent or chronic hyperkalaemia in patients with decreased kidney function. Nutrients.

[23] Gritter M, Rotmans JI, Hoorn EJ (2019). Role of dietary K + in natriuresis, blood pressure reduction, cardiovascular protection, and renoprotection. Hypertension.

[24] van Ypersele de Strihou C (1977). Potassium homeostasis in renal failure. Kidney Int.

[25] Cupisti A, Brunori G, Di Iorio BR (2018). Nutritional treatment of advanced CKD: twenty consensus statements. J Nephrol.

[26] Weiner ID, Wingo CS (1998). Hyperkalemia: a potential silent killer. J Am Soc Nephrol.

[27] Wu X, Zhang W, Ren H (2014). Diuretics associated acute kidney injury: clinical and pathological analysis. Ren Fail.

[28] Saran R, Bragg-Gresham JL, Rayner HC (2003). Nonadherence in hemodialysis: associations with mortality, hospitalization, and practice patterns in the DOPPS. Kidney Int.

[29] Torlén K, Kalantar-Zadeh K, Molnar MZ (2012). Serum potassium and cause-specific mortality in a large peritoneal dialysis cohort. Clin J Am Soc Nephrol.

[30] Redaelli B, Locatelli F, Limido D (1996). Effect of a new model of hemodialysis potassium removal on the control of ventricular arrhythmias. Kidney Int.

[31] Sforzini S, Latini R, Mingardi G (1992). Ventricular arrhythmias and 4-year mortality in haemodialysis patients. Gruppo Emodialisi e Patologie Cardiovascolari. Lancet.

[32] Karaboyas A, Zee J, Brunelli SM (2017). Dialysate potassium, serum potassium, mortality, and arrhythmia events in hemodialysis: results from the dialysis outcomes and practice patterns study (DOPPS). Am J Kidney Dis.

[33] Kovesdy CP, Regidor DL, Mehrotra R (2007). Serum and dialysate potassium concentrations and survival in hemodialysis patients. Clin J Am Soc Nephrol.

[34] Lin C-H, Tu Y-F, Chiang W-C (2013). Electrolyte abnormalities and laboratory findings in patients with out-of-hospital cardiac arrest who have kidney disease. Am J Emerg Med.

[35] Pun PH, Lehrich RW, Honeycutt EF (2011). Modifiable risk factors associated with sudden cardiac arrest within hemodialysis clinics. Kidney Int.

[36] Noori N, Kalantar-Zadeh K, Kovesdy CP (2010). Dietary potassium intake and mortality in long-term hemodialysis patients. Am J Kidney Dis.

[37] Sterns RH, Grieff M, Bernstein PL (2016). Treatment of hyperkalemia: something old, something new. Kidney Int.

[38] Mehrotra R, Himmelfarb J (2013). Dialysis in 2012: could longer and more frequent haemodialysis improve outcomes?. Nat Rev Nephrol.

[39] Vanden Hoek TL, Morrison LJ, Shuster M (2010). Part 12: cardiac arrest in special situations: 2010 American Heart Association Guidelines for Cardiopulmonary Resuscitation and Emergency Cardiovascular Care. Circulation.

[40] Aslam S, Friedman EA, Ifudu O (2002). Electrocardiography is unreliable in detecting potentially lethal hyperkalaemia in haemodialysis patients. Nephrol Dial Transplant.

[41] Nakamura T, Fujisaki T, Miyazono M (2018). Risks and benefits of sodium polystyrene sulfonate for hyperkalemia in patients on maintenance hemodialysis. Drugs R D.

[42] Szerlip HM, Weiss J, Singer I (1986). Profound hyperkalemia without electrocardiographic manifestations. Am J Kidney Dis.

[43] Harel Z, Kamel KS (2016). Optimal dose and method of administration of intravenous insulin in the management of emergency hyperkalemia: a systematic review. PLoS ONE.

[44] Collins AJ, Pitt B, Reaven N (2017). Association of serum potassium with all-cause mortality in patients with and without heart failure, chronic kidney disease, and/or diabetes. Am J Nephrol.

[45] Loutradis C, Tolika P, Skodra A (2015). Prevalence of hyperkalemia in diabetic and non-diabetic patients with chronic kidney disease: a nested case-control study. Am J Nephrol.

[46] Chang AR, Sang Y, Leddy J (2016). Antihypertensive medications and the prevalence of hyperkalemia in a large health system. Hypertension.

[47] Heerspink HJL, Kröpelin TF, Hoekman J (2015). Drug-induced reduction in albuminuria is associated with subsequent renoprotection: a meta-analysis. J Am Soc Nephrol.

[48] Lewis EJ, Hunsicker LG, Clarke WR (2001). Renoprotective effect of the angiotensin-receptor antagonist irbesartan in patients with nephropathy due to type 2 diabetes. N Engl J Med.

[49] Brenner BM, Cooper ME, de Zeeuw D (2001). Effects of losartan on renal and cardiovascular outcomes in patients with type 2 diabetes and nephropathy. N Engl J Med.

[50] Parving H-H, Brenner BM, McMurray JJV (2012). Cardiorenal end points in a trial of aliskiren for type 2 diabetes. N Engl J Med.

[51] Fried LF, Emanuele N, Zhang JH (2013). Combined angiotensin inhibition for the treatment of diabetic nephropathy. N Engl J Med.

[52] Mavrakanas TA, Gariani K, Martin P-Y (2014). Mineralocorticoid receptor blockade in addition to angiotensin converting enzyme inhibitor or angiotensin II receptor blocker treatment: an emerging paradigm in diabetic nephropathy: a systematic review. Eur J Intern Med.

[53] Strippoli GFM, Bonifati C, Craig M (2006). Angiotensin converting enzyme inhibitors and angiotensin II receptor antagonists for preventing the progression of diabetic kidney disease. Cochrane Database Syst Rev.

[54] Epstein M, Reaven NL, Funk SE (2015). Evaluation of the treatment gap between clinical guidelines and the utilization of renin-angiotensin-aldosterone system inhibitors. Am J Manag Care.

[55] Bakris GL, Pitt B, Weir MR (2015). Effect of patiromer on serum potassium level in patients with hyperkalemia and diabetic kidney disease: the AMETHYST-DN randomized clinical trial. JAMA.

[56] Packham DK, Rasmussen HS, Lavin PT (2015). Sodium zirconium cyclosilicate in hyperkalemia. N Engl J Med.

[57] Pitt B, Anker SD, Bushinsky DA (2011). Evaluation of the efficacy and safety of RLY5016, a polymeric potassium binder, in a double-blind, placebo-controlled study in patients with chronic heart failure (the PEARL-HF) trial. Eur Heart J.

[58] Bakris GL, Agarwal R, Chan JC (2015). Effect of finerenone on albuminuria in patients with diabetic nephropathy: a randomized clinical trial. JAMA.

[59] van Deursen VM, Urso R, Laroche C (2014). Co-morbidities in patients with heart failure: an analysis of the European Heart Failure Pilot Survey. Eur J Heart Fail.

[60] (2018) 2017 USRDS annual data report: executive summary. Am J Kidney Dis 71:S1–S8. 10.1053/j.ajkd.2018.01.003

[61] Ponikowski P, Voors AA, Anker SD (2016). ESC guidelines for the diagnosis and treatment of acute and chronic heart failure: the task force for the diagnosis and treatment of acute and chronic heart failure of the European Society of Cardiology (ESC) developed with the special contribution of the Heart Failure Association (HFA) of the ESC. Eur Heart J.

[62] Yancy CW, Jessup M, Bozkurt B (2017). ACC/AHA/HFSA focused update of the 2013 ACCF/AHA guideline for the management of heart failure: a report of the American College of Cardiology/American Heart Association Task Force on Clinical Practice Guidelines and the Heart Failure Society of America. Circulation.

[63] Maggioni AP, Anker SD, Dahlström U (2013). Are hospitalized or ambulatory patients with heart failure treated in accordance with European Society of Cardiology guidelines? Evidence from 12,440 patients of the ESC Heart failure long-term registry. Eur J Heart Fail.

[64] Aldahl M, Jensen A-SC, Davidsen L (2017). Associations of serum potassium levels with mortality in chronic heart failure patients. Eur Heart J.

[65] Pitt B, Zannad F, Remme WJ (1999). The effect of spironolactone on morbidity and mortality in patients with severe heart failure. Randomized aldactone evaluation study investigators. N Engl J Med.

[66] Juurlink DN, Mamdani MM, Lee DS (2004). Rates of hyperkalemia after publication of the randomized aldactone evaluation study. N Engl J Med.

[67] Vardeny O, Wu DH, Desai A (2012). Influence of baseline and worsening renal function on efficacy of spironolactone in patients with severe heart failure: insights from RALES (randomized aldactone evaluation study). J Am Coll Cardiol.

[68] Pitt B, Remme W, Zannad F (2003). Eplerenone, a selective aldosterone blocker, in patients with left ventricular dysfunction after myocardial infarction. N Engl J Med.

[69] Eschalier R, McMurray JJV, Swedberg K (2013). Safety and efficacy of eplerenone in patients at high risk for hyperkalemia and/or worsening renal function: analyses of the EMPHASIS-HF study subgroups (Eplerenone in Mild Patients Hospitalization And SurvIval Study in Heart Failure). J Am Coll Cardiol.

[70] Cooper LB, Lippmann SJ, Greiner MA (2017). Use of mineralocorticoid receptor antagonists in patients with heart failure and comorbid diabetes mellitus or chronic kidney disease. J Am Heart Assoc.

[71] Beldhuis IE, Myhre PL, Claggett B (2019). Efficacy and safety of spironolactone in patients With HFpEF and chronic kidney disease. JACC Heart Fail.

[72] McMurray JJV, Packer M, Desai AS (2014). Angiotensin–neprilysin inhibition versus enalapril in heart failure. N Engl J Med.

[73] Williams B, MacDonald TM, Morant SV (2018). Endocrine and haemodynamic changes in resistant hypertension, and blood pressure responses to spironolactone or amiloride: the PATHWAY-2 mechanisms substudies. Lancet Diabetes Endocrinol.

[74] Zhao D, Liu H, Dong P, Zhao J (2017). A meta-analysis of add-on use of spironolactone in patients with resistant hypertension. Int J Cardiol.

[75] Sim JJ, Bhandari SK, Shi J (2013). Characteristics of resistant hypertension in a large, ethnically diverse hypertension population of an integrated health system. Mayo Clin Proc.

[76] Acharya T, Tringali S, Singh M, Huang J (2014). Resistant hypertension and associated comorbidities in a veterans affairs population. J Clin Hypertens (Greenwich).

[77] De Nicola L, Gabbai FB, Agarwal R (2013). Prevalence and prognostic role of resistant hypertension in chronic kidney disease patients. J Am Coll Cardiol.

[78] Václavík J, Sedlák R, Plachy M (2011). Addition of spironolactone in patients with resistant arterial hypertension (ASPIRANT): a randomized, double-blind, placebo-controlled trial. Hypertension.

[79] Pisoni R, Acelajado MC, Cartmill FR (2012). Long-term effects of aldosterone blockade in resistant hypertension associated with chronic kidney disease. J Hum Hypertens.

[80] Abolghasmi R, Taziki O (2011). Efficacy of low dose spironolactone in chronic kidney disease with resistant hypertension. Saudi J Kidney Dis Transpl.

[81] Agarwal R, Rossignol P, Garza D (2018). Patiromer to enable spironolactone use in the treatment of patients with resistant hypertension and chronic kidney disease: rationale and design of the AMBER study. Am J Nephrol.

[82] Williams B, Mancia G, Spiering W (2018). ESC/ESH Guidelines for the management of arterial hypertension. Eur Heart J.

[83] Carey RM, Calhoun DA, Bakris GL (2018). Resistant hypertension: detection, evaluation, and management: a scientific statement from the american heart association. Hypertension.

[84] Faubel S, Shah PB (2016). Immediate consequences of acute kidney injury: the impact of traditional and nontraditional complications on mortality in acute kidney injury. Adv Chronic Kidney Dis.

[85] Bagshaw SM, Wald R (2017). Strategies for the optimal timing to start renal replacement therapy in critically ill patients with acute kidney injury. Kidney Int.

[86] Libório AB, Leite TT, de Neves FM (2015). AKI complications in critically ill patients: association with mortality rates and RRT. Clin J Am Soc Nephrol.

[87] An JN, Lee JP, Jeon HJ (2012). Severe hyperkalemia requiring hospitalization: predictors of mortality. Crit Care.

[88] Ahmed J, Weisberg LS (2001). Hyperkalemia in dialysis patients. Semin Dial.

[89] Fisch C (1973). Relation of electrolyte disturbances to cardiac arrhythmias. Circulation.

[90] Campese VM, Adenuga G (2011). Electrophysiological and clinical consequences of hyperkalemia. Kidney Int Suppl.

[91] Freeman K, Feldman JA, Mitchell P (2008). Effects of presentation and electrocardiogram on time to treatment of hyperkalemia. Acad Emerg Med.

[92] Long B, Warix JR, Koyfman A (2018). Controversies in management of hyperkalemia. J Emerg Med.

[93] Moulik PK, Nethaji C, Khaleeli AA (2002). Misleading electrocardiographic results in patient with hyperkalaemia and diabetic ketoacidosis. BMJ.

[94] Wang K (2004). Images in clinical medicine. “Pseudoinfarction” pattern due to hyperkalemia. N Engl J Med.

[95] Hung AM, Hakim RM (2015). Dialysate and serum potassium in hemodialysis. Am J Kidney Dis.

[96] Pun PH, Lehrich RW, Honeycutt EF (2011). Modifiable risk factors associated with sudden cardiac arrest within hemodialysis clinics. Kidney Int.

[97] Robert T, Joseph A, Mesnard L (2016). Calcium salt during hyperkalemia. Kidney Int.

[98] Li T, Vijayan A (2014). Insulin for the treatment of hyperkalemia: a double-edged sword?. Clin Kidney J.

[99] McNicholas BA, Pham MH, Carli K (2018). Treatment of hyperkalemia with a low-dose insulin protocol is effective and results in reduced hypoglycemia. Kidney Int Rep.

[100] Wheeler DT, Schafers SJ, Horwedel TA (2016). Weight-based insulin dosing for acute hyperkalemia results in less hypoglycemia. J Hosp Med.

[101] Rossignol P, Legrand M, Kosiborod M (2016). Emergency management of severe hyperkalemia: guideline for best practice and opportunities for the future. Pharmacol Res.

[102] Meaney CJ, Beccari MV, Yang Y, Zhao J (2017). Systematic review and meta-analysis of patiromer and sodium zirconium cyclosilicate: a new armamentarium for the treatment of hyperkalemia. Pharmacotherapy.

[103] Kosiborod M, Peacock WF, Packham DK (2015). Sodium zirconium cyclosilicate for urgent therapy of severe hyperkalemia. N Engl J Med.

[104] Betts KA, Woolley JM, Mu F (2017). The cost of hyperkalemia in the United States. Kidney Int Rep.

[105] Weir MR (2011). Current and future treatment options for managing hyperkalemia. Kidney Int Suppl.

